# *Parabacteroides goldsteinii* Alleviates Intestinal Inflammation in Dextran Sulfate Sodium-Treated Pigs

**DOI:** 10.3390/ani15091231

**Published:** 2025-04-27

**Authors:** Xu Deng, Taozong Guo, Yang He, Shengnan Gao, Jirong Su, Hongbin Pan, Anjian Li

**Affiliations:** Yunnan Provincial Key Laboratory of Animal Nutrition and Feed Science, Faculty of Animal Science and Technology, Yunnan Agricultural University, Kunming 650201, China; dx13354902937@163.com (X.D.); 15925052801@163.com (T.G.); lunahy2022@163.com (Y.H.); gaoshengnan2333@163.com (S.G.); sjr1848766@163.com (J.S.)

**Keywords:** piglet, intestinal inflammation, *Parabacteroides goldsteinii*, intestinal flora

## Abstract

Intestinal inflammation in piglets leads to diarrhea, decreased immune function, and dysbiosis of the gut microbiota. Probiotics are widely recognized and used in the prevention and control of enteritis in piglets. *Parabacteroides goldsteinii* (PG) is a probiotic, and there are few reports on the anti-inflammatory properties of this in piglets. Therefore, this study selected 10 Duroc × Landrace × Yorkshire (DLY) piglets aged 50 days and randomly divided them into a control group (CT group) and an experimental group (PG group) for a 14-day experiment. During days 1–7, the PG group established an inflammation model by gavage with 4% dextran sulfate sodium (DSS). The results showed that DSS increased the content of IL-6 and IL-8 (*p* < 0.05), while it decreased the content of IL-10 in the serum (*p* < 0.05), For days 8–14, the DSS-treated piglets were administered a 7.9 × 10^8^ CFU/mL PG suspension via gavage. The content of IL-6, IL-8, and IL-10 in the piglets did not differ between the CT and PG groups (*p* > 0.05). Some beneficial bacteria, such as *Lactobacillales* and *Butyricimonas*, were significantly enriched in the PG group (*p* < 0.05). The PG group showed lower alpha diversity (*p* < 0.05), and the metabolic pathways exhibited the highest abundance in the KEGG functional prediction analysis. The above studies confirm that PG alleviates intestinal inflammation and changes the gut microbial composition.

## Simple Summary

The effects of *Parabacteroides goldsteinii* on intestinal inflammation and intestinal flora were evaluated. In piglets, where intestinal inflammation was induced by dextran sulfate sodium (DSS), serum inflammatory factors returned to normal levels following intragastric administration of PG, indicating that the inflammation was alleviated. Additionally, beneficial bacteria were significantly enriched in the intestines of the piglets in the PG group. These positive effects confirm that PG can alleviate intestinal inflammation in piglets and has anti-inflammatory properties.

## 1. Introduction

Inflammatory bowel disease (IBD) involves various pathogenic factors, such as abnormal gut microbiota and host immunity, ultimately leading to symptoms such as bloody stools and weight loss [1,2]. Under intestinal microbiota disorder, IBD susceptibility genes are overexpressed, exacerbating the inflammatory response [3]. The gut microbiota of piglets is immature and vulnerable to environmental factors [4]. In early-weaned piglets, weaning stress leads to changes in the morphology and function of the small intestine, disrupts gut barrier function, and causes post-weaning diarrhea [5]. This seriously threatens the health of piglets and increases the chance of reduced growth rates, weight loss, and death, thereby increasing breeding costs and reducing economic benefits [6]. Gut microbiota stability plays a key role in improving colitis. The establishment of a healthy gastrointestinal environment is particularly important for piglet growth and disease resistance [7]. Antibiotics can effectively control diseases in livestock and poultry and improve growth performance [8]. However, due to the overuse of antibiotics, many bacterial species have developed drug resistance and antibiotic-resistant genes (ARGs), which are regarded as emerging pollutants that threaten the environment and human health [9]. Probiotics, as alternatives to antibiotics, have multiple health benefits for livestock and poultry [10,11]. They can prevent diarrhea and irritable bowel syndrome, participate in immune regulation, regulate the gut microbiota, and improve feed efficiency and growth performance [12]. Therefore, probiotic supplementation is an effective method for relieving colitis.

Currently, the most studied probiotics in the livestock and poultry industry are lactic acid bacteria, *Bifidobacterium*, and yeast [13,14]. These probiotics interact with immune cells to maintain the immune balance in the gastrointestinal tract. They also produce beneficial microbial metabolites, enhance the integrity of the intestinal mucosa, reduce pathogen colonization, and alleviate intestinal inflammatory responses [15]. *Parabacteroides* is an obligate anaerobic Gram-negative bacterium and a core member of the gut microbiota. It is important for host health [16]. With the development of metagenomics, an increasing number of *Parabacteroides* species have been isolated and identified. The predominant species are *Parabacteroides goldsteinii* and *Parabacteroides distasonis*, respectively [17]. These species exert probiotic functions that maintain host gut homeostasis, including regulating host metabolism and the immune system, secreting metabolites, and alleviating inflammation [18,19,20].

Previous research has indicated that *Parabacteroides goldsteinii* (PG) can mitigate obesity and improve chronic obstructive pulmonary disease (COPD) and may play a role in reducing inflammation [18,21,22]. In this study, a model of colitis was established in weaned piglets, and the therapeutic effect of orally administered *P. goldsteinii* on colitis was explored. Additionally, the impact of *P. goldsteinii* treatment on the intestinal microbiota of piglets was investigated. This study aimed to provide fundamental evidence for the future application of *P. goldsteinii* in alleviating colitis in weaned piglets and to identify candidate strains for use as probiotics to relieve colitis.

## 2. Materials and Methods

### 2.1. Preparation of P. goldsteinii

*Parabacteroides goldsteinii* (Microbe Division, Institute of Physical and Chemical Research, Japan) was cultured according to instructions. Upon the completion of cultivation, *P. goldsteinii* was centrifuged. Subsequently, the pellet was resuspended in normal saline to prepare the *P. goldsteinii* suspension (7.9 × 10^8^ CFU/mL).

### 2.2. Pigs and Experimental Design

This experiment was approved by the Animal Care and Use Committee of Yunnan Agricultural University (Approval number: 202103056). Ten healthy 50-day-old Duroc × Landrace × Yorkshire (DLY) pigs were divided into the control (CT) and experimental (PG) groups. The pigs were fed the same diet (Table 1) according to the National Research Council [23], and had free access to clean water (Nipple drinkers). All pigs were vaccinated according to standard procedures. For the PG treatment (5 pigs), 200 mL of 4% dextran sulfate sodium (DSS) was administered by gavage on the first day, and then 100 mL of 4% DSS was administered by gavage daily from the second to the seventh day. For the CT treatment (5 pigs), the same dose of normal saline was administered by gavage from the first to the seventh day. The DSS dose was selected using the method described by Zhao et al. (2022) [24]. From days 8 to 14 of the PG treatment, 10 mL of *P. goldsteinii* suspension was administered by gavage, and pigs in the CT treatment group were administered the same dose of normal saline by gavage. At the end of the experiment, 10 mL blood samples were collected from anterior vena cava and centrifuged to obtain serum. Fecal samples were collected for 16S rRNA sequencing analysis. The serum and fecal samples were stored at −70 °C prior to analysis.

### 2.3. Detection of IL-1β, IL-6, IL-8, and IL-10 in the Serum of Pigs

The serum IL-1β, IL-6, IL-8, and IL-10 expression levels were detected using an interleukin-1β ELISA Assay Kit, Interleukin-6 ELISA Assay Kit, Interleukin-8 ELISA Assay Kit, and Interleukin-10 ELISA Assay Kit (Nanjing Jiancheng Bioengineering Institute, Nanjing, China) as per the manufacturer’s instructions.

### 2.4. 16S rRNA Sequencing

The 16S rRNA sequencing was performed as described by Hu et al. (2024) [25]. Briefly, the libraries of 16S rRNA were constructed from the amplified DNA by the SMRTbell prep kit 3.0 (Pacific Biosciences, Menlo Park, CA, USA) according to the manufacturer’s instructions. Amplicon sequencing was performed by Shanghai Biozero Biotechnology Co. Ltd. (Shanghai, China).

After sequencing, the raw reads were processed using SMRT Link Analysis software version 11.0. UPARSE software (version 7.1, http://drive5.com/uparse/, accessed on 1 December 2023) was used to cluster operational taxonomic units (OTUs) at a similarity threshold of 98.65%. Subsequently, UCHIME was used to identify and eliminate the chimeric sequences.

The RDP Classifier (https://lcsciences.com/documents/sample_data/16S_sequencing/src/html/top1.html, accessed on 1 December 2023) was used to analyze the phylogenetic relationships of each 16S rRNA gene sequence by comparing them against the Silva (SSU132) 16S rRNA database, with a confidence threshold of 70%. Each sequence was annotated for species classification using the RDP Classifier (https://lcsciences.com/documents/sample_data/16S_sequencing/src/html/top1.html, version 2.2) in comparison with the Silva 16S rRNA database (v138) with a comparison threshold set at 80%.

To obtain species-classification information corresponding to each OTU, the UCLUST algorithm was adopted for the taxonomic analysis of the representative OTU sequences. The community composition of each sample was statistically analyzed at various taxonomic levels, including the phylum, genus, and species levels.

The α-diversity of the species (including ACE, Chao1, and Shannon indices) and the beta-diversity through principal component analysis (PCA) were determined. Based on the high-quality reads, Tax4Fun (version 1.0) was used to predict the functional categories of the KEGG homologs.

### 2.5. Statistical Analysis

The data were analyzed using the Student’s *t*-test in SPSS 22.0. All data are presented as the mean ± standard error. Differences were considered statistically significant at *p* < 0.05.

## 3. Results

### 3.1. Effect of DSS on the Serum Levels of Inflammatory Factors in Piglets

**Figure 1:**
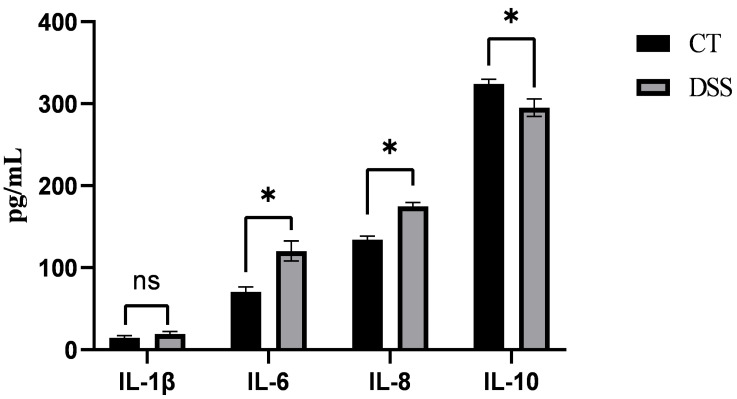
Serum content of IL-6, IL-8, and IL-10 after DSS treatment; The asterisk “*” indicates a significant difference (*p* > 0.05), while “ns” signifies no significant difference (*p* > 0.05).

After gavage with DSS in piglets, compared with those in the CT group, the serum levels of IL-6 and IL-8 significantly increased (*p* < 0.05), whereas the level of IL-10 significantly decreased (*p* < 0.05), indicating that the colitis model in piglets was successfully established (Figure 1).

### 3.2. Effect of PG on the Serum Levels of Inflammatory Factors in DSS-Induced Inflammatory Piglets

**Figure 2:**
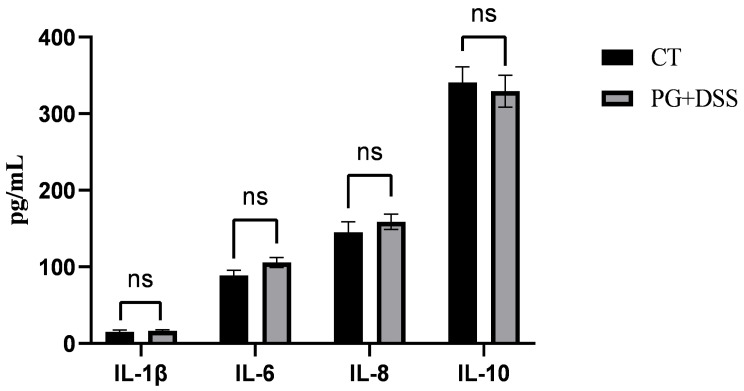
Serum content of IL-6, IL-8, and IL-10 after PG treatment; “ns” signifies no significant difference (*p* > 0.05).

After gavage with the *P. goldsteinii* suspension in DSS-induced inflammation piglet models, there were no significant differences in the serum levels of IL-6, IL-8, and IL-10 compared with those in the CT group (*p* > 0.05), and the levels of IL-6 and IL-8 increased in the PG group (Figure 2).

### 3.3. OTU Clustering Analysis

**Figure 3:**
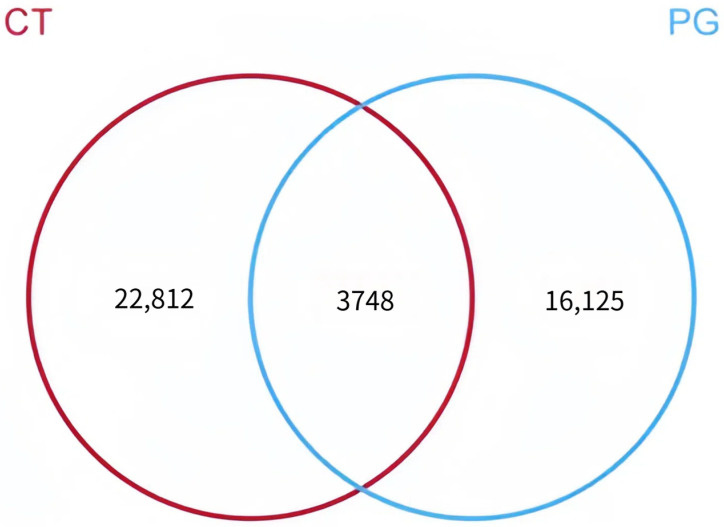
Venn diagram of OTU distribution between groups.

The numbers of OTUs in the CT and PG groups were 26,560 and 19,873, respectively. Among these, 3478 shared OTUs were identified. The CT group had 22,812 unique OTUs, and the PG group had 16,125 unique OTUs (Figure 3).

### 3.4. Alpha Diversity Analysis

**Figure 4:**
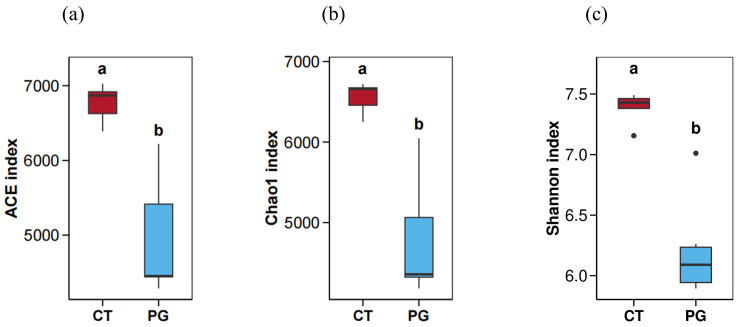
Alpha diversity map. (**a**) ACE index; (**b**) Chao1 index; (**c**) Shannon index; Significant differences (*p* < 0.05) are denoted by the distinct letters a and b.

As shown in Figure 4, the ACE, Chao1, and Shannon indices of the CT group were significantly higher than those of the PG group (*p* < 0.05).

### 3.5. Microbial Species Abundance and Taxonomic Statistics

#### 3.5.1. Phylum-Level Species Composition

**Figure 5:**
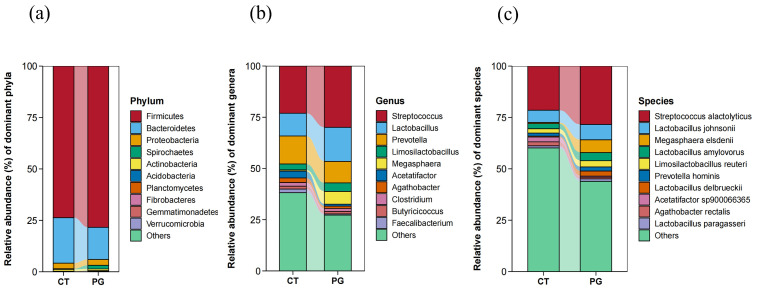
(**a**) Microbial composition of fecal microbiota in piglets at the phylum level; (**b**) Microbial composition of fecal microbiota in piglets at the genus level; (**c**) Microbial composition of fecal microbiota in piglets at the species level.

At the phylum level, the fecal microbiota in both groups were mainly composed of Firmicutes, Bacteroidetes, Proteobacteria, Spirochaetes, and Actinobacteria (Figure 5a).

#### 3.5.2. Genus-Level Microbiota Composition

At the genus level, the fecal microbiota in both groups were mainly composed of *Streptococcus*, *Lactobacillus*, *Prevotella*, *Megasphaera*, and *Limosilactobacillus*. However, no significant differences were observed in the dominant genera between the two groups (Figure 5b).

#### 3.5.3. Species-Level Microbiota Composition

At the species level, the fecal microbiota in both groups were mainly composed of *Streptococcus alactolyticus*, *Lactobacillus johnsonii*, *Megasphaera elsdenii*, *Lactobacillus amylovorus*, and *Prevotella hominis*. However, no significant differences were observed in the dominant genera between the two groups (Figure 5c).

### 3.6. LEfSe Analysis

**Figure 6:**
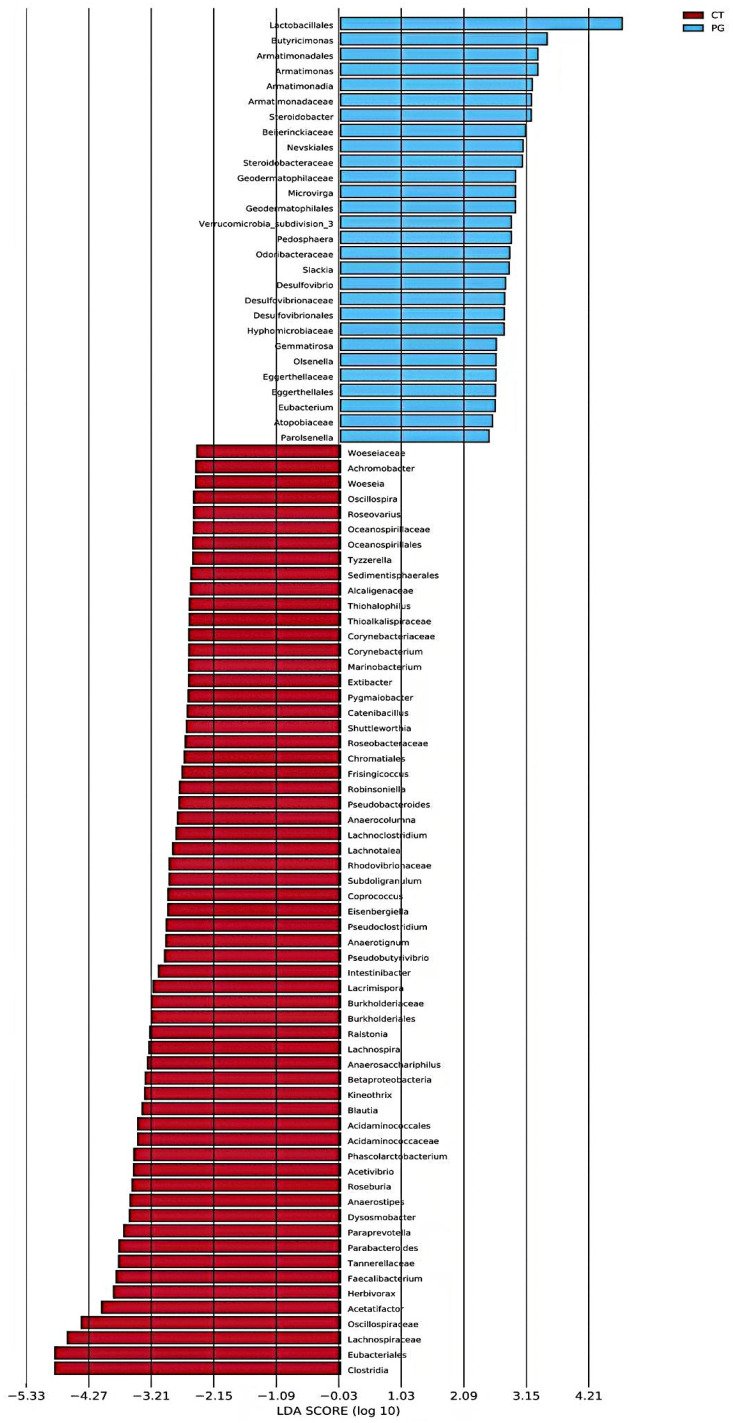
Histogram of LDA value distribution in piglet fecal microbiota.

LEfSe analysis revealed that the CT group was significantly enriched in *Clostridia*, *Eubacteriales*, and *Lachnospiraceae*, whereas the PG group was significantly enriched in *Lactobacillales*, *Butyricimonas*, and *Armatimonadales* (Figure 6).

### 3.7. KEGG Pathway Functional Analysis

**Figure 7:**
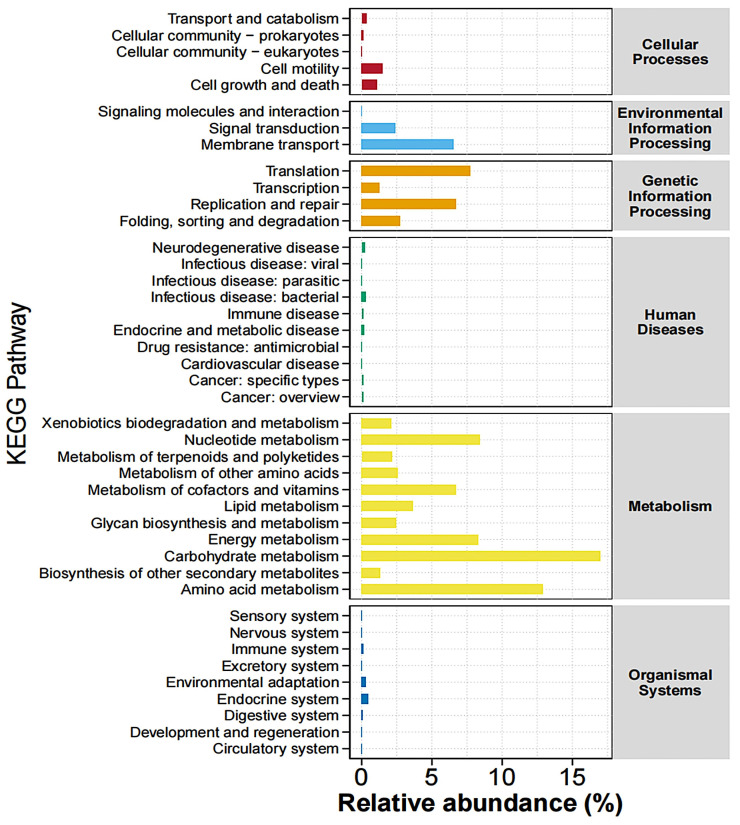
Abundance statistics of KEGG pathway. Different colors represent distinct pathways at the first hierarchical level of KEGG.

Among the enriched KEGG pathways, the majority of the pathways belonged to “metabolism”, including carbohydrate metabolism, amino acid metabolism, xenobiotic biodegradation and metabolism, nucleotide metabolism, metabolism of terpenoids and polyketides, metabolism of other amino acids, metabolism of cofactors and vitamins, lipid metabolism, glycan biosynthesis and metabolism, energy metabolism, and biosynthesis of other secondary metabolites (Figure 7).

## 4. Discussion

Inflammation is a defense mechanism that involves the migration of white blood cells to damaged tissues and the production of various cytokines [26]. IL-6 is rapidly produced in response to stress, tumorigenesis, and acute inflammatory reactions and regulates immune responses and acute-phase reactions [27]. IL-8 exacerbates inflammatory reactions by recruiting and activating neutrophils and promoting their infiltration into inflamed tissues, which is a key factor in several inflammatory diseases, such as rheumatoid arthritis and IBD [28]. IL-10 suppresses pro-inflammatory responses, reduces tissue damage, and exerts anti-inflammatory effects. Moreover, IL-10 is a major cytokine in intestinal regulation [29]. DSS induces intestinal inflammation and microbiota dysbiosis, mimicking the acute and chronic inflammatory processes of UC. Yuan et al. (2023) found that the levels of pro-inflammatory cytokines, such as IL-6, TNF-α, and IL-1β, were significantly increased in the serum of DSS-treated mice, while IL-10 levels were markedly reduced, indicating the successful establishment of an inflammatory model [30]. In this study, DSS was administered to piglets via oral gavage to induce colitis. Compared with those in the CT group, the contents of IL-6 and IL-8 were increased, whereas the contents of IL-10 were decreased in the DSS treatment. This indicated that the DSS-induced colitis model was successfully established in piglets. Therefore, we investigated the anti-inflammatory activity of PG suspensions administered by gavage in piglets with colitis.

IBD is a microbiota-related disease caused by abnormal interactions between microbes and the host due to various factors, such as intestinal microbiota dysbiosis, impaired intestinal epithelial barrier function, and weakened immune function, leading to inflammatory responses [31]. Probiotics can help reduce intestinal inflammation and maintain gut health [32]. Metabolites of probiotics, such as organic acids, particularly acetic and butyric acids, have strong bactericidal effects against Gram-negative bacteria, thereby reducing tissue damage [33]. Kverka et al. (2010) found that the oral administration of *Parabacteroides distasonis* to DSS-treated mice significantly reduced the levels of pro-inflammatory cytokines and increased serum antibody levels, thereby alleviating inflammation [34]. *Parabacteroides goldsteinii* (PG) is an emerging next-generation probiotic with potential anti-inflammatory properties. Lai et al. (2022) discovered that the lipopolysaccharide (LPS) of PG could reduce the levels of the pro-inflammatory cytokines IL-1β and TNF-α in mice with colitis, mitigate inflammatory damage, and maintain the integrity of the intestinal epithelium, demonstrating its anti-inflammatory effects in mice [18]. In this study, after successfully establishing a colitis model in piglets, a PG suspension was administered via gavage to the colitis piglets. Compared with those in the CT group, there were no significant differences in the levels of the cytokines in the treatment group, indicating that the levels of inflammatory cytokines returned to normal and colitis in the piglets was alleviated. This finding is consistent with those of previous studies, suggesting that PG exerts anti-inflammatory effects on intestinal inflammation in piglets.

Probiotics alter the diversity of host gut microbiota. The results of the 16S rRNA analysis in this study showed that the gut microbiota diversity of DSS-induced inflammatory piglets decreased after gavage with the PG suspension, indicating that PG can modify the gut microbiota. Zhu et al. (2022) found that the diversity of a probiotic group was significantly reduced in mice with colitis, and the beneficial effects were attributed to the reduced growth of pro-inflammatory bacteria in the gut [35]. The composition of the gut microbiota in animals is highly complex, with Firmicutes and Bacteroidetes being the most abundant phyla, followed by Proteobacteria. Interactions among the microbes belonging to these phyla and between these microbes and the host provide beneficial functions to the host [36]. The gut microbiota is in a relatively balanced state, and any imbalance can lead to diseases in the host. Zhao et al. (2022) demonstrated that *Bacteroides*, *Desulfovibrio*, and *Streptococcus*, which are associated with inflammation, were enriched in Yorkshire pigs following DSS treatment [24]. Overgrowth of these pathogenic bacteria leads to the excessive production of pathogen-associated molecular patterns (PAMPs), which, when recognized by the host immune cells, trigger inflammatory responses, resulting in intestinal damage and promoting the development of subsequent diseases such as metabolic endotoxemia, obesity, dyslipidemia, hepatic steatosis, and IBD [37]. The reduced microbial diversity in the PG group, along with the increase in inflammation, may be due to the probiotic functions of PG, which inhibit the growth of harmful bacteria.

LEfSe differential analysis showed that beneficial bacteria, such as *Lactobacillales* and *Butyricimonas*, were enriched in the PG group (*p* < 0.05). *Lactobacillus* spp. are the most common probiotics. They can activate superoxide dismutase and reduce the release of inflammatory factors, thereby alleviating intestinal damage and reducing the incidence of colorectal cancer and tumors caused by IBD [38,39]. *Lactobacillales* can also improve intestinal barrier function and reduce the incidence and recurrence rate of UC by upregulating the expression of 5-hydroxytryptamine transporter (SERT) and transforming growth factor (TGF-β) [40]. The increase in CD4^+^T cells in the lamina propria of the colon may be related to an increase in the abundance of *Lactobacillales*, maintaining the TH2-type immune response and immune tolerance pattern and preventing excessive inflammatory responses [41]. Additionally, metabolites produced by *Lactobacillales*, such as bacteriocins and organic acids, inhibit the growth of other bacteria, which further protects the body [42,43,44]. Lactic acid reduces the pH of the intestinal lumen. Bacteroidetes are relatively sensitive to pH, whereas Firmicutes and *Bifidobacterium* have stronger tolerances [45]. The significant enrichment of *Lactobacillales* in the PG group, accompanied by a decrease in the abundance of Bacteroidetes, may be due to the inhibitory effect of organic acid metabolites produced by *Lactobacillales*.

*Butyricimonas* can break down complex polysaccharides, such as cellulose and hemicellulose, to produce short-chain fatty acids (SCFAs), such as butyrate and propionate [46]. As the main by-products of microbial fermentation, they can provide energy for the intestinal epithelium, promote the proliferation of epithelial cells, lower the intestinal pH, and have excellent antibacterial and anti-inflammatory activities, thereby playing an important role in maintaining intestinal homeostasis and suppressing intestinal inflammation [47,48]. *Butyricimonas* has beneficial effects on lipid metabolism and immune function [49].

Intestinal bacteria may exert their effects through other metabolic pathways. KEGG biological function prediction analysis revealed that carbohydrate and amino acid metabolism pathways had the highest enrichment levels. The microbiota can regulate these metabolic processes to produce a variety of intermediate metabolites that influence the host immune system [50,51]. PG alleviates colitis in piglets by enriching these pathways. Under pathophysiological conditions, an imbalance in the gut microbiota composition occurs along with the enrichment of harmful bacteria. However, in the PG group, the abundance of various beneficial microorganisms increased, and inflammation was alleviated. This indicates that the addition of PG reversed the imbalance in the gut microbiota to a stable state, thereby exerting an anti-inflammatory function by regulating the composition of the gut microbiota.

## 5. Conclusions

PG alleviates the intestinal inflammatory response in piglets by reducing the production of pro-inflammatory factors, such as IL-6 and IL-8, and increasing the levels of the anti-inflammatory factor IL-10. The underlying mechanism may be that the PG bacterial solution increases the abundance of beneficial bacteria, such as *Lactobacillales* and *Butyricimonas*, in DSS-induced piglets. This study indicates that PG can be used as a beneficial bacterium to prevent colitis in pigs.

## Figures and Tables

**Table 1:** Basal diet composition and nutrient level.

Component	Content (%)	Nutritional Level ^b^	Content (%)
Corn	62.00	Digestible energy (MJ/kg)	13.61
soybean meal	26.60	Crude protein	18.01
wheat bran	5.50	Ca	0.77
Soybean oil	1.25	Total P	0.62
CaHPO_4_·2H_2_O	1.30	Lysine	1.29
Limestone	1.00	Met+Cys	0.74
lysine	0.50		
methionine	0.15		
NaCl	0.70		
Premix ^a^	1.00		
Total	100		

^a^ Premix supplied per kg diet: vitamin A, 5000 IU; vitamin D_3_, 900 IU; vitamin E, 45 IU; vitamin B_1_, 12 mg; choline, 0.4 g; pantothenic acid, 15 mg; folic acid, 0.38 mg; Fe, 80 mg; Cu, 18 mg; Zn, 100 mg; I, 0.35 mg; Se, 0.27 mg; ^b^ In the nutritional level, digestible energy is a theoretical value, while the remaining components are measured values.

## Data Availability

The raw sequencing data have been deposited in the China National GeneBank Sequence Archive (CNSA) of the China National GeneBank DataBase (CNGBdb) with the accession number CNP0005846.
